# Price transparency in the Dutch market-based health care system: did price dispersion for similar hospital services reduce over time?

**DOI:** 10.1007/s10198-025-01759-6

**Published:** 2025-02-22

**Authors:** Frédérique Franken, Rudy Douven, Stéphanie van der Geest, Marco Varkevisser

**Affiliations:** 1 Erasmus School of Health Policy & Management (ESHPM), Erasmus University Rotterdam, Rotterdam, The Netherlands; 2 Erasmus Centre for Health Economics Rotterdam (EsCHER), Rotterdam, The Netherlands

**Keywords:** Price dispersion, Hospital pricing, Price transparency, Insurer-provider negotiations, C23, D40, D82, I11, I13

## Abstract

In market-based health care systems, insurers negotiate prices of hospital care products with providers. While few countries disclose these negotiated prices, in 2016, the Dutch government required the disclosure of insurer-provider negotiated prices for hospital products up to €885 – the maximum deductible in the Netherlands – to enhance price transparency. This aimed to increase price awareness among and price transparency for consumers, insurers, and providers, fostering price competition. We study if price dispersion for relatively homogeneous hospital care products decreased post-publication, resulting in price convergence. We used negotiated price data from three major Dutch health insurers on over 200 hospital products. Using descriptive statistics and linear regression, with the coefficient of variation (a measure of dispersion) regressed on the year, we examined the development of price dispersion and the occurrence of price convergence. Price dispersion for the studied sample of hospital products decreased by an average of 29% between 2016 and 2022. This decrease was not accompanied by a price level increase that was larger than expected based on general inflation. Regression analysis showed a significant negative association between year and the coefficient of variation, indicating price convergence. These findings support our hypothesis that price dispersion decreased after mandatory price disclosure. The government mandate potentially increased awareness of largely unexplainable price differences for products priced below €885, encouraging insurers and providers to reduce these through the negotiation process. The observed price convergence likely benefits patients, as it results in less random out-of-pocket payments across providers for the same hospitals products.

## Introduction

In countries with a market-based health care system, insurers typically negotiate contracts and prices with health care providers [1]. It is assumed that insurers, spurred on by competition on premiums, attempt to negotiate uniformly low prices across all providers of the same health care product. However, a substantial body of research has shown large price dispersion for similar hospital care products. Cooper et al. [2] demonstrated, for example, that 50% of the variation in health care spending among United States (US) hospitals was the result of price dispersion. This large amount of price dispersion was also observed for nearly homogeneous care services, suggesting that the dispersion was not solely driven by differences in case mix or quality [2, 3]. Prices diverge substantially not only between health care providers but also between insurers [4]. Remarkably, price dispersion exists even within insurers, with negotiated prices for commercial plans that are two to three times greater than those for Medicare Advantage plans for the same insurer, provider and service [5]. In the Netherlands, Douven et al. [6] studied the dispersion in prices across hospitals negotiated by one of the largest Dutch health insurers. They demonstrated that price dispersion was substantial, for 30% of the hospital product prices were at least 20% higher or lower compared to the mean. Among other things, the authors mentioned a difference in costing methods, uncertainty about underlying costs or the strategic adjustment of product prices to generate the lump sum payment as possible explanations [6].

Previously considered commercially sensitive, some countries now publish insurer-provider negotiated prices with the aim of increasing transparency and decreasing price dispersion. In the US, for example, negotiated prices have become increasingly available to the public due to the Hospital Price Transparency Rule mandated by the Centers for Medicare and Medicaid Services (CMS) in January 2021 [7]. In the Netherlands, the government mandated the publication of the provider-insurer negotiated hospital product prices up to €885 in 2016 [8].

These price transparency initiatives have the potential of increasing awareness among consumers, insurers, and providers regarding price differences for the same health care product. They decrease information asymmetry and may therefore increase competition on prices, which could reduce price dispersion across providers of the same health care product. Any remaining price dispersion is then likely to result predominantly from differences in case-mix or quality. The two channels through which increased price transparency may theoretically lead to less price dispersion can be explained as follows.

Firstly, price transparency initiatives may facilitate informed patient decision-making, enabling cost-conscious patients to choose lower-priced providers, in particular for ‘shoppable’ hospital services for which patients incur out-of-pocket costs. This may narrow the hospital product price range and lower the price level through increased provider-competition [9]. A scoping review by Zhang et al. [10] showed, however, that uptake of price transparency information by patients was weak. Among many potential explanations, information asymmetry with respect to the pricing system and the health care services needed may be an important barrier preventing patients from using price information. The health care system may be complex to navigate, patients may not know what to look for [11], and they have been shown to rely heavily on the referring physician [12]. Moreover, insurance coverage largely renders the search for lower prices useless, as patients only have to pay a relatively small share out-of-pocket. The beneficial effects of increased price transparency through this first channel are therefore likely to be modest. If price information is at all used, there is also research that indicates that patients choose higher-priced providers as they associate higher prices with higher quality [13]. While this may lead to decreased price dispersion, it may result in increased overall price levels if it strengthens the leverage of providers to negotiate higher prices.

Secondly, price transparency may lead to decreased price dispersion via the interaction between insurers and providers. Increased price transparency may encourage insurers and providers to alter prices in response to increased competition [9, 14]. Although insurers could previously compare their own negotiated prices across all providers, and providers could compare their own prices negotiated with all insurers, the price publication mandate now allows them to compare prices negotiated by their direct competitors as well. Both providers and insurers can use the prices published by their competitors as a benchmark, comparing them to the prices they have negotiated themselves and using the average price per product across providers and/or insurers as a yardstick.

Increased benchmark price information is likely to result in less price dispersion. Whether it will over time also lead to a lower average price level, however, depends on the relative bargaining power of the insurers and providers in the health care market. Both insurer and provider market power influence the extent to which price information is used to negotiate higher or lower prices. On the one hand, insurers with sufficient market power may leverage price information to negotiate *lower* prices with relatively high-priced providers [15, 16]. On the other hand, relatively lower-priced providers with sufficient market power can use the information to negotiate *higher* prices with the insurers [17, 18].

In this paper, we study the amount of price dispersion for relatively homogeneous hospital care products costing up to €885 (e.g. outpatient clinic visits or small procedures) across providers in the Dutch hospital market since the 2016 price transparency mandate. Unlike Douven et al. [6], who studied only one insurer in the first year of the mandate’s implementation, we examined the negotiated hospital product prices from three insurers, covering multiple years. This allows us to explore whether the increased availability of benchmark price information is associated with a decrease in price dispersion across providers over time. In addition, we investigate if the potential downward trend in price dispersion differed between health insurers, which may indicate a difference in their ability or inclination to use the price information. To this end, we collected price data from three major Dutch insurers on more than 200 hospital products between 2016 and 2022. We analysed the data using descriptive statistics, graphs, and linear regression.

This paper is structured as follows. First, we describe the Dutch institutional context. Then, we describe our data and the empirical methodology. We proceed to summarise the results and conclude this paper with a discussion of the findings.

## Institutional context

In the Netherlands, private health insurers are expected to act as prudent buyers of accessible and high-quality hospital care on behalf of their enrolees, for whom buying a government defined basic benefit package is mandatory since the implementation of the Health Insurance Act (HIA) in 2006 [19–21]. All hospital services are classified into Diagnosis-Treatment Combinations (DTCs). These DTCs are similar to Diagnosis-Related Groups (DRGs), but also cover outpatient care and medical specialist remuneration. DTCs can be divided into two segments. The ‘A-segment’ contains DTCs for which prices are administered and mostly consists of high-complexity, relatively expensive hospital products or products for which the government considered price competition undesirable. The ‘B-segment’ contains DTCs for which prices are determined through negotiations between health insurers and providers. The share of the B-segment DTCs, for which price competition is allowed, has gradually increased over the years and accounted for more than 80% of Dutch hospital spending in 2021 [22]. Each year, health insurers and providers negotiate prices of individual B-segment DTCs, in combination with a (maximum) lump sum payment encompassing a global budget for all DTCs. In the Netherlands, hospital care is provided by hospitals and independent treatment centres (ITCs). While there is a large overlap in the care products provided by hospitals and ITCs, the latter focuses primarily on elective and lower-complexity care products in one or a few specialties (amounting to 6.1% of hospital care revenues in 2022) [23]. For both hospitals and ITCs, all care products are classified into DTCs.

The government mandate that requires health insurers to publish the insurer-provider negotiated prices for hospital products up to €885 went into effect in 2016 [8]. According to public data, the mandate affected between 25 and 28% of healthcare spending (calculated as the spending on DTCs with an average national price below €885 as a percentage of total spending) [24].^1^ The mandate aimed to decrease random price dispersion and encourage price differentiation (for the same hospital product) at the individual hospital level that merely corresponded to variation in case mix and/or quality. Additionally, the increased price transparency intended to benefit patients. €885, a sum of the mandatory deductible of €385 and the maximum voluntary deductible of €500, is the maximum out-of-pocket price for patients (per year). Given the chosen price-publication cut-off of €885, the DTCs for which prices were disclosed are relatively inexpensive and part of basic hospital care. Therefore, relative homogeneity and a limited difference in provider quality is expected. Decreased random price dispersion for DTCs up until this price may therefore reduce random out-of-pocket payments for patients.

Although mandatory, for unknown reasons, the price transparency mandate was not actively enforced nor strictly monitored by the government, resulting in a changing number of health insurers publishing their yearly insurer-provider negotiated prices for hospital products [25]. Nevertheless, due to the price transparency mandate, both insurers and providers may have gained knowledge about the prices their competitors negotiated in the past years. This may have led to converging prices for similar hospital products, while the extent to which this contributed to a general price increase or decrease is unclear.

## Methods

### Data sources and set of hospital care products studied

For this study, we used insurer-provider negotiated DTC prices from three major Dutch health insurers (CZ, VGZ, and Zilveren Kruis) with a combined market share of over 70% in 2022 [26]. While VGZ (indicated as insurer A) and Zilveren Kruis (insurer B) provided data on their negotiated prices for hospital products in Excel files on their website, CZ (insurer C) opted for an online tool that could be used by their enrolees to inquire about and compare providers’ prices for a specific hospital product. Upon request, insurer C also provided us with their prices in Excel format. A fourth major health insurer, Menzis, did not publish negotiated prices on its website. We did not receive the relevant data upon request via e-mail. Considering that the health insurers for which data was available represented a considerable market share, we did not actively pursue data from the smaller health insurers (whose individual market shares did not exceed 4.5% in 2022) [26].

Data for insurers A and C was available for the years 2016 and 2019–2022, while data for insurer B was available for the years 2020–2022, which led us to analyse two separate datasets. The data included the DTC code and its description, the reimbursement code, the negotiated price, and the name of the provider with which the price had been negotiated. Insurers B and C also reported the providers’ AGB (Algemeen GegevensBeheer*)* code, a unique and time-invariant provider identifier. The online Vektis AGB code register was used to create a translation table containing the AGB codes and the various provider spellings encountered in the data [27]. The providers in insurer A’s datasets were consequently matched to their respective AGB codes. The reimbursement code specified for every price in the dataset allowed us to include only DTCs with freely negotiable prices (the B-segment) covered by the basic benefit package. Only DTCs included in the basic benefit package affect the deductible, as all other care is not reimbursed through the mandatory insurance.

The government’s specified cut-off point (€885) was interpreted differently by the insurers. While all insurers reported all prices below €885, insurer C also reported the negotiated prices above €885 for DTCs for which they had negotiated at least one price below €885. Insurer A used a similar approach but reported prices above €885 as ‘higher than €885’ instead of the actual price. The prices reported by insurer B were limited to those below €885, resulting in uncertainty regarding whether the absence of certain providers was due to non-contracting or non-disclosing of prices above €885. To prevent non-random missing data, we chose to include only those DTCs priced below €885 for all providers in all included years. Moreover, we included only those DTCs negotiated by all insurers in all years. Finally, we included per DTC only those insurer-provider combinations present in all included years. The data primarily consisted of common DTCs such as outpatient clinic visits, diagnostic tests, small procedures or day treatment.

Data on patient volumes per DTC (on a national level), measured by the total number of claims during a year, as well as on mean prices per DTC (on a national level) were obtained through the publicly available Open DIS database [24].

Since we aimed to observe dispersion in DTC prices negotiated by multiple insurers, our data was identified by two panels – one across multiple DTCs and one across multiple insurers – in addition to the time panel, resulting in multidimensional data. We constructed a panel identifier by grouping DTC and insurer, hereby enabling two-dimensional panel data analysis. Two balanced panel datasets were constructed, based on the available data. Dataset 1 included data from insurers A and C during the period 2016 and 2019–2022, while dataset 2 included all three insurers during 2020–2022. While they partly overlap, dataset 1 is richer in terms of years and dataset 2 is richer in terms of insurers. Hence, we used both datasets to examine both price dispersion across providers – including all hospital types as well as ITCs – and across insurers.

### Empirical methodology

In this paper, price dispersion across providers of the same hospital product is calculated as the coefficient of variation (CV), measuring the price dispersion per DTC for the individual insurers. The CV, defined as the standard deviation divided by the mean price multiplied by 100, is a relative measure of dispersion. The use of a ratio renders this measure unitless, making it well suited for the comparison of price dispersion among DTCs with substantially varying mean prices. Descriptive statistics and graphs were used to visualise price dispersion as well as its development over the years.

To test for a statistically significant trend in price dispersion during the study period as well as any statistically significant differences in price dispersion (trends) across insurers, we estimated the following linear regression model:

$$\begin{aligned}{CV}_{it}&=\:{\beta\:}_{1}{Year}_{t}\cr&\quad+{\beta\:}_{2}{Insurer}_{i}+{\beta\:}_{3}{Insurer}_{i}*{Year}_{t}\cr&\quad+\:{\beta\:}_{4}{Volume}_{it}+{v}_{it}\end{aligned}$$

where $\:{CV}_{it}$ represents our measure of price dispersion for every DTC-insurer combination $\:i$ in year $\:t$, reflecting the variation in prices that an insurer has negotiated with providers of a specific DTC in a given year. To test for a time effect over the included years – our primary interest – we included $\:n$-1 dummy variables for the year $\:t$ in which the price dispersion was observed ($\:{Year}_{t}$). We included dummy variables for the insurer with which the prices for DTC-insurer combination $\:i$ were negotiated ($\:{Insurer}_{i}$) to control for time-invariant insurer-specific effects (e.g. overall negotiating skills or inclination to use benchmark price information). Moreover, we included an interaction term $\:{Insurer}_{i}*{Year}_{t}$ to examine whether a potential price dispersion trend over time differed per insurer. To examine if price dispersion differed based on patient volume (e.g. because the potential budget impact is higher for high-volume products, warranting special attention from insurers during the negotiation process), we controlled for patient volume per DTC $\:i$ in year $\:t$, measured by the total number of times a DTC was reimbursed per year on a national level ($\:{Volume}_{it}$). $\:{v}_{it}$ denotes the composite error $\:{u}_{it}+{c}_{i}$, where $\:{u}_{it}$ is the individual-variant and time-variant idiosyncratic error and $\:{c}_{i}$ the time-invariant heterogeneity [28]. To check for the robustness of our empirical findings, we estimated a pooled ordinary least squares (OLS) model using a simple linear regression, a fixed-effects (FE) model in which we assume a correlation between the explanatory variables and, and a random-effects (RE) model in which $\:{x}_{it}$ and $\:{c}_{i}$, are assumed to be not correlated.

## Results

### Descriptive results

Dataset 1, including prices from insurers A and C, comprised of 205 DTCs in the period 2016 and 2019–2022. Dataset 2, including prices from all three insurers (A, B, and C), comprised of 250 DTCs in the period 2020–2022. Table 1 provides the descriptive statistics per year for both datasets. The mean nominal price level (the mean of nominal prices paid at the time of the insurer-provider transaction that have not been adjusted for inflation) across all DTC-insurer combinations included in dataset 1 increased with 7.40% from €277.53 in 2016 to €298.08 in 2022. These prices were similar to the mean prices at the national level – with the difference between the national mean price and mean price in the study sample ranging from 0.22 to 2.72% – suggesting that the data we collected is representative of the prices negotiated by all Dutch insurers. The mean annual CV across all DTC-insurer combinations declined from 19.32 in 2016 to 13.77 in 2022, resulting in an overall 28.73% decrease. The extent of the price dispersion decreases most in the first three years after the price publication mandate, with 4.17 percentage points, amounting to a decrease of 21.58%. We find a smaller decrease of 1.38 percentage points (9.11%) between 2019 and 2022. The insurers negotiated prices with a mean of 47 providers per DTC, meaning that each DTC-insurer combination in the data includes on average 47 different negotiated prices. By construction of the dataset, this number is equal for all years. The average number of patients per included DTC-insurer combination ranged from 19,000 to 20,700 per year. The mean nominal price across all DTC-insurer combinations included in dataset 2 was slightly higher compared to dataset 1 and increased with 6.01% from €304.13 in 2020 to €322.41 in 2022. As expected, the average annual CV in dataset 2 was similar to dataset 1, decreasing every subsequent year from 14.86 in 2020 to 13.30 in 2022 (a 10.50% decrease). Prices were negotiated with a larger mean number of 56 providers. The larger number of included DTCs in dataset 2 may be explained by the fact that it exclusively covered the period 2020–2022, which was characterised by a lower CV and, consequently, less price dispersion compared to earlier years (Table 1). This increased the probability of insurers having negotiated a price below €885 for all providers and likely contributed to a larger number of DTCs matching our selection criterion. Figure 3a and b in the Appendix show the distribution of the CV for both datasets.

**Fig. 1:**
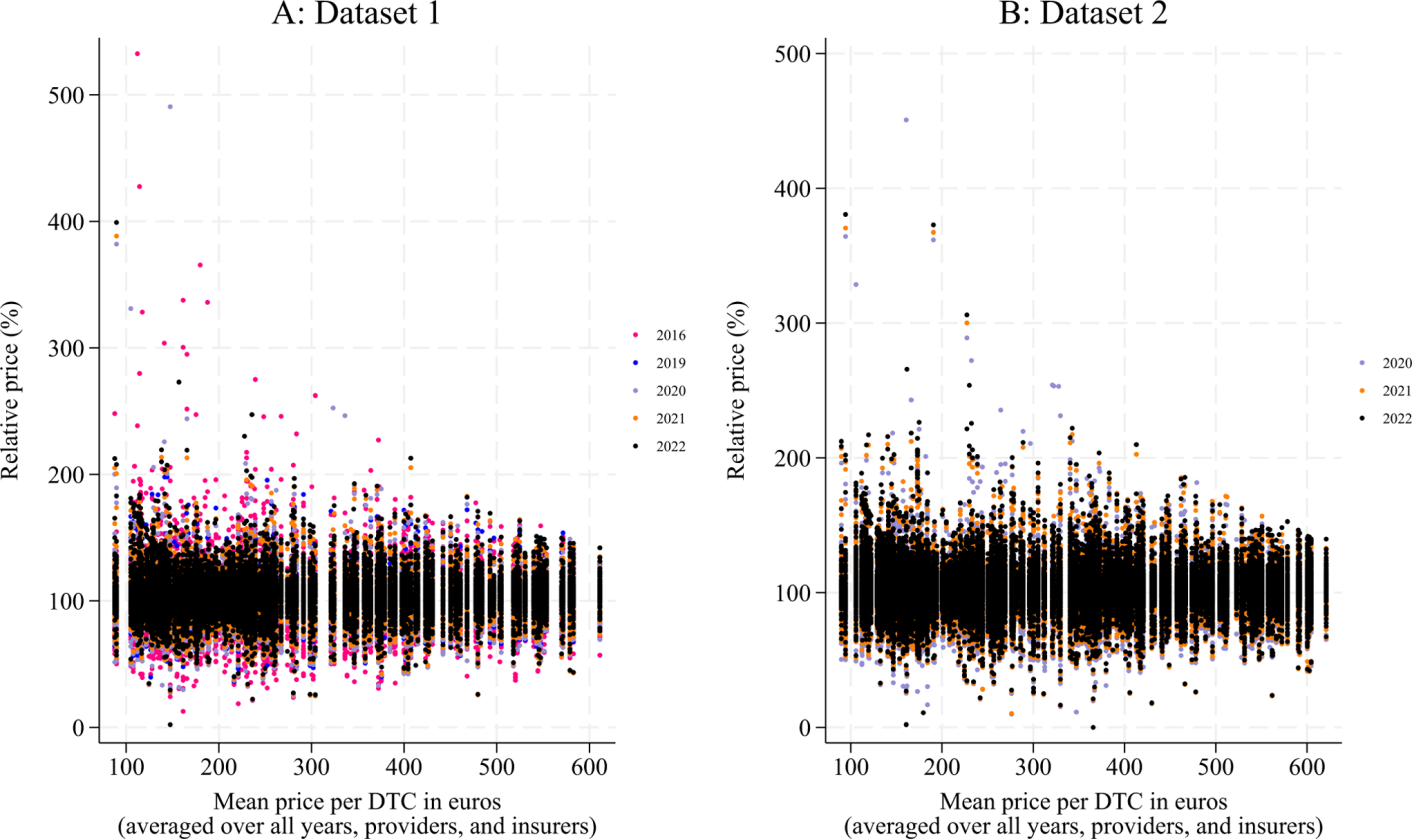
Relative prices calculated as the insurer-provider negotiated prices per year as a percentage of the corresponding mean nominal Diagnosis-Treatment Combination (DTC) price for that year on the y-axis ranked by the corresponding mean nominal DTC price over all years, providers, and insurers on the x-axis

In Fig. 1a and b, each dot represents a relative price for a specific DTC in a year calculated as the insurer-provider negotiated price as a percentage of the corresponding mean nominal DTC price at the national level. On the x-axis, each relative price is plotted against the corresponding mean nominal DTC price over all years, providers, and insurers. Similar to the descriptive statistics in Table 1, the figures suggest that the price dispersion has decreased over the years, with less deviation from the mean. Nevertheless, in 2022, approximately 15% of the prices in both datasets still deviated 20% or more from the mean price.

**Fig. 2:**
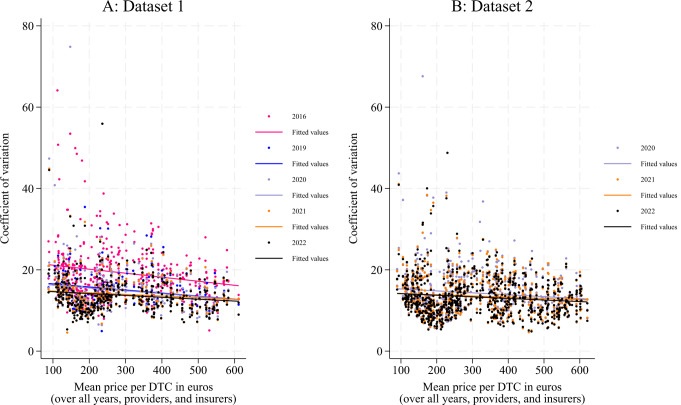
The development of price dispersion. The y-axis shows the coefficient of variation (CV) per Diagnosis-Treatment Combination (DTC)-insurer combination per year, ranked by the corresponding mean nominal DTC price over all years, providers, and insurers on the x-axis. A regression line using the CV as the outcome and the mean nominal DTC price as the only explanatory variable was fitted for each of the years using ordinary least squares

The development of price dispersion is illustrated in Fig. 2a and b. Similar to Fig. 1a and b, the x-axis ranks the DTCs from less to more expensive. The y-axis shows the CV, with one observation per DTC-insurer combination per year. To visualise the development of price dispersion, a regression line (using the mean nominal DTC price as the only explanatory variable) was fitted for each of the years using OLS. The figures show that for both datasets, the regression lines from later years are below the ones from earlier years, indicating a CV that decreases over the years, but particularly between 2016 and 2019. The figures also show that the CV is higher for the less expensive DTCs, compared to the more expensive DTCs.

**Table 1:** Descriptive statistics for dataset 1 and dataset 2

	2016		2019		2020		2021		2022		
	Mean	St. dev	Mean	St. dev	Mean	St. dev	Mean	St. dev	Mean	St. dev	
**Dataset 1 (2 insurers**,** 205 DTCs)**											
National price (€)	276.91	131.58	282.62	135.38	287.45	137.73	296.72	142.45	306.42	147.04	
Price in study sample (€)	277.53	132.80	278.61	132.51	281.22	134.03	289.10	138.42	298.08	142.72	
CV (%)^a^	19.32	6.74	15.15	5.50	14.95	5.40	14.02	4.21	13.77	4.68	
Patient volume (n, per 10000)	1.95	2.59	2.07	2.66	1.90	2.41	1.97	2.50	1.97	2.51	
**Dataset 2 (3 insurers**,** 250 DTCs)**											
National price (€)					311.73	146.35	321.71	151.31	332.20	156.02	
Price in study sample (€)					304.13	140.63	312.96	145.01	322.41	149.56	
CV (%)^a^					14.86	4.93	13.53	4.71	13.30	4.84	
Patient volume (n, per 10000)					1.80	2.64	1.86	2.73	1.88	2.82	

DTC = Diagnosis-Treatment Combination, CV = Coefficient of Variation ^a^ = computed as the average of the individual CVs per DTC

### Estimation results

To test whether the decrease in price dispersion during the study period was statistically significant, we estimated a linear regression model.^2^ Tables 2 and 3 present our FE estimation results for dataset 1 and 2 respectively. As explained above, we estimated models using pooled OLS, FE, and RE. For each of these estimators, we estimated a model with only the variable of primary interest and a model with control variables. Residual plots as well as quantile-quantile plots were made and the Breusch-Pagan test was performed to test for heteroskedasticity, which proved to be present. Hence, cluster-robust standard errors were used for all models to account for heteroskedasticity (and serial correlation) [29]. We discuss the FE estimation results below. As robustness checks, Tables 4 and 5 in the Appendix show the results for the pooled OLS and RE models. The coefficients estimated by these models for the dummy variables indicating the year, our variable of primary interest, are consistent with those estimated using the FE model.

In general, Tables 2 and 3 demonstrate that the year variables are significantly associated with the CV for both datasets. Table 2 shows that, compared to 2016, the CV was significantly lower in 2019, 2020, 2021, and 2022 at the 5% level, showing an average decrease in the CV of 4.524 percentage points in 2022 compared to 2016. The extent of price dispersion decreases most in the first three years after the price publication mandate, with over 3.5 percentage points. Using the FE model, the insurer dummy variable was omitted from the analysis as it is time-invariant. The significant coefficients for the interaction term (except for insurer A*Year 2020) indicate that the association between year and CV is influenced by the insurer. The decrease in price dispersion is significantly greater for insurer A compared to insurer C, with coefficients ranging from − 1.146 to -2.485. This suggests that, all else equal, insurer A was able to negotiate prices that vary less between providers compared to those of insurer C. Patient volume was not significantly associated with the CV.

**Table 2:** Fixed-effects regression results dataset 1

	(1)	(2)
	Fixed effects	Fixed effects
Year 2019	-4.168^***^	-3.574^***^
	(0.287)	(0.282)
Year 2020	-4.369^***^	-4.004^***^
	(0.285)	(0.273)
Year 2021	-5.300^***^	-4.054^***^
	(0.297)	(0.274)
Year 2022	-5.552^***^	-4.524^***^
	(0.313)	(0.344)
Insurer A		-
Insurer A * Year 2019		-1.146^*^
		(0.571)
Insurer A * Year 2020		-0.745
		(0.568)
Insurer A * Year 2021		-2.485^***^
		(0.582)
Insurer A * Year 2022		-2.047^**^
		(0.618)
Patient volume		-0.170
		(0.360)
Constant	19.32^***^	19.65^***^
	(0.225)	(0.706)
Observations	2050	2050
Within *R*^*2*^	0.340	0.357
Between *R*^*2*^		0.033
Overall *R*^*2*^	0.123	0.150

Standard errors in parentheses: * *p* < 0.05, ** *p* < 0.01, *** *p* < 0.001

**Table 3:** Fixed-effects regression results dataset 2

	(1)	(2)
	Fixed effects	Fixed effects
Year 2021	-0.751^***^	-0.026
	(0.086)	(0.024)
Year 2022	-0.977^***^	-0.490^**^
	(0.105)	(0.177)
Insurer A		-
Insurer B		-
Insurer A * Year 2021		-2.300^***^
		(0.226)
Insurer B * Year 2021		0.060^*^
		(0.029)
Insurer A * Year 2022		-1.872^***^
		(0.285)
Insurer B * Year 2022		0.332
		(0.192)
Patient volume		0.327
		(0.170)
Constant	14.28^***^	13.69^***^
	(0.060)	(0.291)
Observations	2247	2247
Within *R*^*2*^	0.086	0.207
Between *R*^*2*^	0.000	0.034
Overall *R*^*2*^	0.007	0.008

Standard errors in parentheses: * *p* < 0.05, ** *p* < 0.01, *** *p* < 0.001

Table 3 indicates that, while the 0.490 percentage point decrease in price dispersion between 2020 and 2022 is significant, the 0.026 percentage point decrease between 2020 and 2021 is not. For dataset 2, the association between year and CV is also influenced by the insurer. The decrease in price dispersion is significantly greater for insurer A in 2021 and 2022, with coefficients of -2.300 and − 1.872. The 2021 price dispersion for insurer B, however, is slightly higher compared to insurer C. Again, patient volume was not significantly associated with the CV.

## Conclusions and discussion

In this study, we examined the development of price dispersion and the potential occurrence of price convergence in the Dutch hospital care sector after the increase in price transparency due to the mandatory publication of negotiated insurer-provider prices up to €885 (i.e., the maximum annual deductible level). The Dutch government mandated the publication of these prices by the insurers in 2016, with the aim of decreasing seemingly random price differences. Decreasing the price differences for products up to €885 would automatically lead to less random out-of-pocket payments. We used publicly available price data from three major Dutch health insurers (CZ, VGZ, and Zilveren Kruis) to calculate the coefficient of variation for a set of relatively homogeneous and inexpensive hospital products. Since the included health insurers jointly hold a market share of around 70%, and the mean prices in our data approximated the national mean prices, our data was likely a representative sample of the insurer-provider negotiated prices in the Netherlands. Two datasets were constructed to examine if price dispersion decreased in the years following the price publication mandate.

Despite the increased price transparency after 2016, price dispersion for hospital products up to €885 across providers remained substantial, with 15% of prices still deviating 20% or more from the mean price. However, price dispersion for the studied sample of hospital products decreased by an average of almost 29% between 2016 and 2022. This decrease in price dispersion was not accompanied by a price level increase larger than expected based on the general inflation rate of 20% or the hospital tariff inflation rate of 17% between 2016 and 2022 [30, 31]. The regression estimators for the included years indicated that the decrease in the coefficient of variation was statistically significant. These results indicate that for the hospital products in our sample, the insurers have been able to reduce the price dispersion without having to accept an increase in the average hospital price level.

While we can conclude that price dispersion has significantly decreased between 2016 and 2022, we are not able to determine the underlying causes and mechanisms. Increased price awareness among insurers as well as providers resulting from the price transparency mandate is likely to have influenced insurer-hospital negotiated prices, but the exact mechanism remains unclear. Moreover, due to the way in which prices were published, we were not able to include data on all products (partly) priced under €885. Additionally, price information for hospital products exceeding €885 is not disclosed, prohibiting the comparison of price dispersion trends for products below and above this cut-off. Consequently, we are unable to explore whether a decrease in price dispersion for products below €885 may be accompanied by an increase in price dispersion (or hospital price level) for products above €885, which could suggest cross-subsidization. This provides an opportunity for further research. In the absence of a larger-than-expected price increase, price convergence may theoretically have resulted in quality skimping to reduce costs. As previously mentioned, however, we expect the difference in quality to be moderate for the lower-complexity products included in our datasets. Nevertheless, we did not have access to quality outcome data for the included care products and we were consequently unable to analyse whether quality changed after the price transparency mandate. Lastly, the use of a DTC-insurer panel and a time-panel – rather than a DTC-provider panel and a time panel – because of data availability impeded us from analysing the relationship between provider attributes and price dispersion. For the same reason, even if information on products priced above €885 had been available, we would not have been able to analyse the extent to which different provider types engage in cross-subsidization.

Overall, we conclude that since 2016, the intended price convergence for similar hospital services with prices up to €885 can indeed be observed, unaccompanied by a price level increase that is larger than expected. This is likely to have resulted in less random out-of-pocket payments across providers for the same hospital products, thereby benefitting patients.

## Appendix

**Fig. 3:**
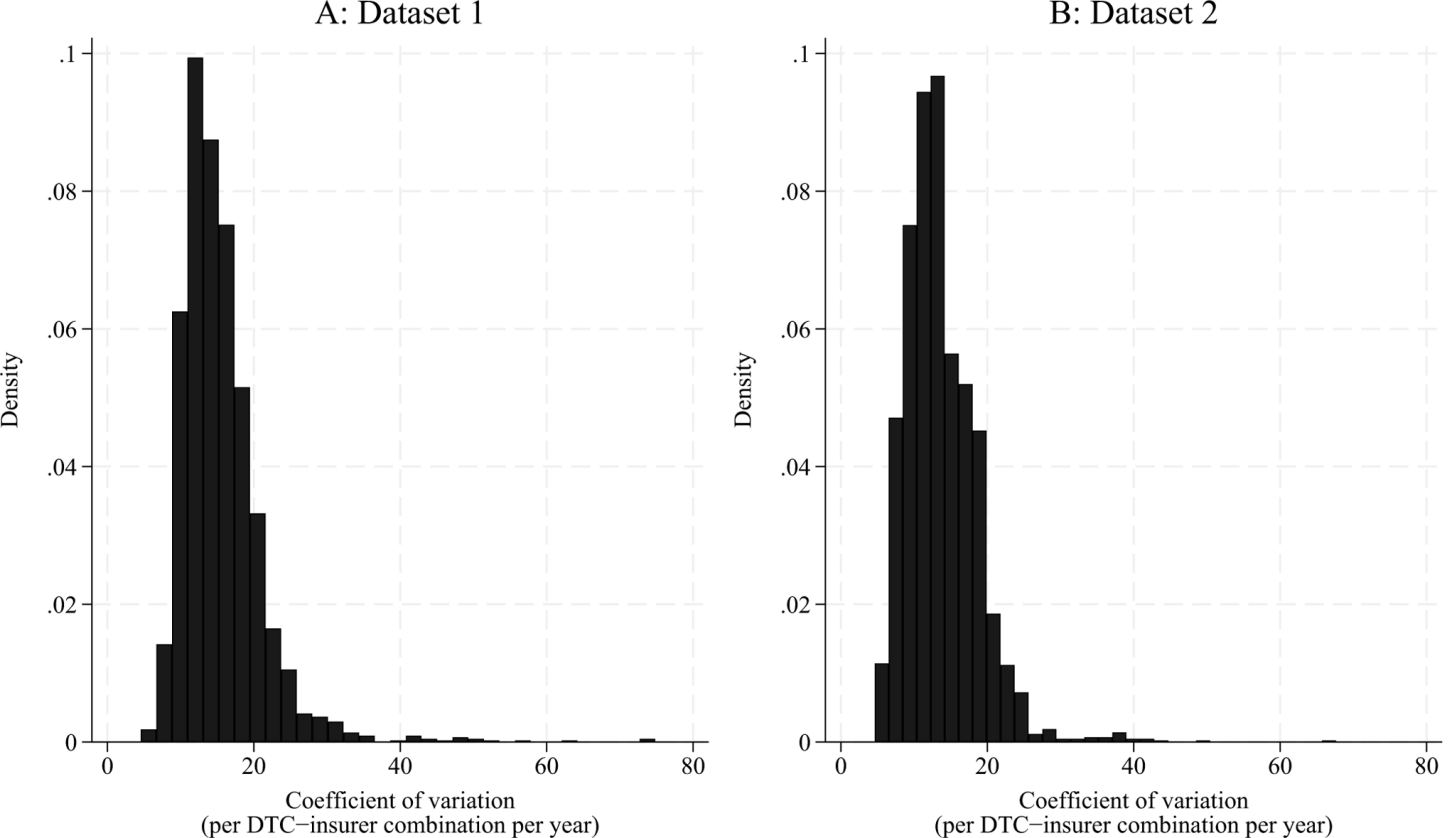
The distribution of the coefficient of variation (per Diagnosis-Treatment Combination (DTC)-insurer combination per year) shown in histograms

**Table 4:** Pooled ordinary least squares and random-effects regression results dataset 1

	(1)	(2)	(3)	(4)
	Pooled OLS	Pooled OLS	Random effects	Random effects
Year 2019	-4.168^***^	-3.545^***^	-4.168^***^	-3.547^***^
	(0.375)	(0.279)	(0.287)	(0.279)
Year 2020	-4.369^***^	-4.015^***^	-4.369^***^	-4.015^***^
	(0.375)	(0.271)	(0.285)	(0.271)
Year 2021	-5.300^***^	-4.049^***^	-5.300^***^	-4.049^***^
	(0.375)	(0.272)	(0.297)	(0.273)
Year 2022	-5.552^***^	-4.517^***^	-5.552^***^	-4.518^***^
	(0.375)	(0.344)	(0.313)	(0.344)
Insurer A		0.681		0.681
		(0.652)		(0.653)
Insurer A * Year 2019		-1.146^*^		-1.146^*^
		(0.572)		(0.572)
Insurer A * Year 2020		-0.745		-0.745
		(0.568)		(0.568)
Insurer A * Year 2021		-2.485^***^		-2.485^***^
		(0.581)		(0.581)
Insurer A * Year 2022		-2.047^**^		-2.047^**^
		(0.619)		(0.619)
Patient volume		-0.409***		-0.393^***^
		(0.083)		(0.079)
Constant	19.32***	19.78***	19.32^***^	19.74^***^
	(0.265)	(0.346)	(0.333)	(0.342)
Observations	2050	2050	2050	2050
Adjusted *R*^*2*^	0.121	0.165		
Within *R*^*2*^			0.000	0.357
Between *R*^*2*^			0.000	0.056
Overall *R*^*2*^			0.123	0.164

Standard errors in parentheses: * *p* < 0.05, ** *p* < 0.01, *** *p* < 0.001

**Table 5:** Pooled ordinary least squares and random-effects regression results dataset 2

	(1)	(2)	(3)	(4)
	Pooled OLS	Pooled OLS	Random effects	Random effects
Year 2021	-0.751^**^	0.012	-0.751^***^	0.010
	(0.259)	(0.021)	(0.086)	(0.021)
Year 2022	-0.977^***^	-0.444^**^	-0.977^***^	-0.447^*^
	(0.259)	(0.175)	(0.105)	(0.175)
Insurer A		0.730		0.731
		(0.469)		(0.470)
Insurer B		-3.789^***^		-3.788^***^
		(0.384)		(0.384)
Insurer A * Year 2021		-2.300^***^		-2.300^***^
		(0.226)		(0.226)
Insurer B * Year 2021		0.060^*^		0.060^*^
		(0.028)		(0.028)
Insurer A * Year 2022		-1.87^***^		-1.872^***^
		(0.285)		(0.286)
Insurer B * Year 2022		0.332		0.332
		(0.192)		(0.192)
Patient volume		-0.222^*^		-0.190^*^
		(0.086)		(0.079)
Constant	14.28^***^	15.70^***^	14.28^***^	15.64^***^
	(0.183)	(0.287)	(0.200)	(0.282)
Observations	2247	2247	2247	2247
Adjusted *R*^*2*^	0.007	0.131		
Within *R*^*2*^			0.000	0.204
Between *R*^*2*^			0.000	0.124
Overall *R*^*2*^			0.007	0.131

Standard errors in parentheses: * *p* < 0.05, ** *p* < 0.01, *** *p* < 0.001

## Footnotes

1 Note that the share of spending on DTCs with average prices below €885 changed from 28.3% in 2016 to 28.0% in 2017 and 25.9% in 2022. Additionally, the share of spending on DTCs with average prices between €800 and €885 changed from 2.7% in 2016 to 2.5% in 2022. Hence, the policy change did not result in a significant shift of DTCs to above the threshold (e.g., to prevent price publication).

2 As a robustness check, we also used the quartile coefficient of dispersion as outcome measure instead of the CV. For our variables of interest (year dummies), this did not alter the findings presented.
